# “Do My Friends Only Like the School Me or the True Me?”: School Belonging, Camouflaging, and Anxiety in Autistic Students

**DOI:** 10.1007/s10803-024-06668-w

**Published:** 2025-01-08

**Authors:** Elizabeth Atkinson, Sarah Wright, Henry Wood-Downie

**Affiliations:** 1 West Sussex Educational Psychology Service, Bridge House, Barrington Road, Goring-By-Sea, Worthing, BN12 4FP UK; 2 Doctorate in Educational Psychology, University of Southampton, Highfield, Southampton S017 1BJ UK

**Keywords:** Autism, School belonging, Camouflaging, Masking, Anxiety, Mixed methods

## Abstract

The prevalence of autistic students in schools is continuously increasing. Typically, the social and sensory differences associated with autism can make the school environment difficult to manage. Autistic students are more likely to experience mental health difficulties than their non-autistic peers. It is therefore important to listen to autistic people about their educational experiences and explore ways that the environment could be changed to reduce anxiety. *Purpose.* The current research explores whether the relationship between school belonging and anxiety in secondary-aged autistic students is mediated by camouflaging. *Methods.* An anonymous online survey was completed by 72 autistic students attending mainstream schools in the UK and Ireland. The survey included questionnaires about school belonging (simple sense of belonging scale), anxiety (ASC-ASD) and camouflaging traits (CAT-Q). Further, the survey included open-ended questions about environmental factors related to school belonging and camouflaging. Qualitative content analysis was used to interpret answers to open ended questions. *Results.* The results found that, as predicted, camouflaging did mediate the relationship between school belonging and anxiety. Categories were created for each question, for example, ‘the school environment’ and ‘acceptance and understanding’. *Conclusion. S*ocial relationships; individual factors; the environment and adaptations, and acceptance and understanding, influence students’ sense of belonging.

**Supplementary Information:**

The online version contains supplementary material available at 10.1007/s10803-024-06668-w.

## Autism, Anxiety, and Mainstream Schools

Autistic people experience differences with sensory processing and social communication (Kirby et al., 2022; Milton, 2012). The prevalence of autism is consistently increasing, and recent government data estimates that nearly 2% of all children in the UK are autistic (Dell’Osso et al. 2022). Approximately 70% of autistic students in the UK and Ireland attend mainstream schools (Dell’Osso et al. 2022; National Autistic Society, 2021; Department of Health, 2018). It has been argued that mainstream school provides social and educational benefits that are enabling for some autistic students (Horgan et al., 2022). Despite this, the environment can prove challenging for autistic students, and research has found that they experience higher levels of anxiety in school than their non-autistic peers (Hebron & Humphrey, 2014; van Steensel et al., 2011; Mesa & Hamilton, 2022), with these difficulties being linked to negative outcomes, including poorer quality of life (Adams et al., 2019), classroom performance (den Houting et al., 2022), school non-attendance (Adams, 2022), and increased suicide ideation (Shtayermman, 2020).

Given the increasing number of autistic students in mainstream schools, it is important to further explore mechanisms behind their increased anxiety levels, while also acknowledging that anxiety may not be inherent in autism. The challenges faced by autistic individuals can be attributed to a mismatch between their unique characteristics and the school environment (Mandy, 2019; Lai & Baron-Cohen, 2015). Research has identified several factors that contribute to anxiety experienced by autistic students in school, including uncertainty, social demands, bullying experiences, and the sensory environment (Horgan et al., 2022; Hebron & Humphrey, 2014; Costley et al., 2021).

## Camouflaging

One factor that is prevalent in the literature relevant to the mental health of autistic people is camouflaging (Cook et al., 2021; Ross et al., 2023). There is no agreed definition of camouflaging (also referred to as masking and adaptive morphing) and further work is needed to explore autistic people’s preferred terminology (Lawson, 2020; Cook et al., 2021; Lai et al., 2021). In the current research, the term ‘camouflaging’ will be used to describe a range of strategies and behaviours that autistic people employ to cope in a neurotypical world (Hull et al., 2017; Cook et al., 2021). Camouflaging can involve hiding or suppressing autistic characteristics (e.g., stimming); copying the behaviours of others who are deemed socially competent; and developing techniques to adopt a non-autistic communication style (Hull et al., 2017). For example, an autistic student in school may use eye-contact despite this feeling unnatural, cover up their need for support in class, or learn scripts to engage with their peers. It has been consistently found that camouflaging is more prevalent in females assigned at birth, and camouflaging may be one possible contributory factor to the late diagnosis of autistic females (McQuaid et al., 2022; Halsall et al., 2021). However, it is noteworthy that camouflaging is not an exclusively female behaviour and has been reported by autistic people of all genders (Ross et al., 2023).

While it could be argued that most people adapt their behaviours to a degree, it has been found that autistic camouflaging requires a level of effort greater than typical “reputation management” (Hull et al., 2017, p. 2520; Cage et al., 2022). Indeed, it has been commonly reported that camouflaging can take a toll on autistic people and their identity, and these compensatory behaviours have been associated with anxiety, depression, and suicidality in the adult population (Cook et al., 2021; Hull et al., 2017; Cassidy et al., 2018; Cage & Troxell-Whitman, 2019). Due to the high prevalence rates of anxiety in autistic secondary aged students (Costley et al., 2021), and inconsistent research findings about camouflaging and general well-being/depression (Cage & Troxell-Whitman, 2019; Perry et al., 2022), the current study will focus primarily on anxiety.

Camouflaging behaviours have been found to start in childhood and develop over time (Ross et al., 2023). Despite most research focussing on adults, there is growing body of evidence exploring the impact of camouflaging in children and adolescents (Cook et al., 2021; Halsall et al., 2021). For example, in two qualitative studies autistic girls have reported that camouflaging caused them a high level of anxiety within school, especially when they were unsure if it had been successful (Halsall et al., 2021; Cook et al., 2018). In a study of over 700 autistic children, Ross et al. (2023) found that camouflaging was positively correlated with internalising symptoms (e.g., anxiety and depression), further evidencing this relationship.

Some autistic people always camouflage, however, for many, it is situation dependent (Hull et al., 2017; Cage & Troxell-Whitman, 2019). Autistic girls have reported that they stop camouflaging when they return home from school, and camouflaging behaviours may be more prevalent in mainstream than specialist settings (Cook et al., 2018; Halsall et al., 2021; Tierney et al., 2016). Given the relationship between camouflaging and anxiety, it is important to look to factors related to the mainstream school context that affect whether autistic students of all genders feel they can ‘take off the mask’; one such concept is school belonging.

## Sense of School Belonging

Psychological literature posits belonging as a need of “all humans in all cultures”, that is key to emotional well-being, and necessary for achieving self-actualisation (Baumeister & Leary, 1995, p. 499; Bowlby, 1969; Maslow, 1968). Indeed, Baumeister and Leary (1995) describe that humans are innately motivated to find and maintain a feeling of belonging. Schools provide a unique and multi-systemic opportunity for pupils to experience belonging, and there has been a wealth of research recognising the importance of belonging in the school context (Arslan et al., 2020).

There are many conceptions of school belonging (also referred to as school connectedness, relatedness, and engagement; Craggs & Kelly, 2018). It is a multidimensional construct related to a student’s affiliation to their school, and includes behavioural and emotional elements (Allen et al., 2021; Arslan et al., 2020). In one commonly cited definition, Goodenow and Grady (2010, p. 61) explain that school belonging is the amount that pupils feel “personally accepted, respected, included, and supported by others”. More recently, in a meta-synthesis of research into school belonging, Craggs and Kelly (2018; p. 1442) conceptualised school belonging as “feeling safe to be yourself in and through relationships in the school setting”.

Research has found that sense of school belonging is associated with better well-being and mental health in both autistic and non-autistic individuals, and that good connectedness to school can protect against mental health difficulties including anxiety (Shochet et al., 2006; Arslan et al., 2020; Benner et al., 2017). Belonging is especially important during adolescence, when priorities and expectations change, and identity is formed (Allen & Kern, 2017). It therefore seems particularly pertinent that autistic students, who are at elevated risk of anxiety, are supported to feel a connection to their schools (Hebron & Humphrey, 2014; Hebron, 2018).

It has been found that autistic students in mainstream schools experience lower levels of school connectedness than their peers (Hebron, 2018; Porter & Ingram, 2021). Friendships, teacher relationships, and understanding/acceptance have been found to be key for developing a sense of school belonging (Craggs & Kelly, 2018; Allen et al., 2021; Allen & Kern, 2017). Autistic students at secondary schools have reported having negative interactions with their teachers, and the sensory environment and unpredictability can lead them to feel excluded (Horgan et al., 2022). Further, due to their different communication styles and the stigma of their diagnosis, autistic students have reported experiencing a high level of bullying and social isolation (Cook et al., 2018; Horgan et al., 2022). All these factors are likely to decrease their sense of school belonging.

There is preliminary evidence that some autistic students who feel a low sense of school belonging camouflage their difficulties. Research in females at secondary school reflects findings in the adult population; that camouflaging is used as a means of forming relationships, trying to fit in, and seeking a sense of belonging, often after previous social rejection (Hull et al., 2017; Tierney et al., 2016; Halsall et al., 2021; Cook et al., 2018). In a qualitative study exploring sense of belonging in autistic females in secondary school, Myles et al. (2019) found that autistic students were motivated to belong, and often adapted their behaviour so that they could experience a sense of belonging. It seems that if students do not feel safe to *be themselves* then they may hide their difficulties in a bid to be *accepted and respected*, which may increase anxiety. Indeed, in a survey of 108 secondary aged females with special educational needs, Porter and Ingram (2021, p. 69) found that students felt that perceived lack of belonging meant that they hid their “quirky bits”, which increased anxiety levels in school. It is important that this relationship is quantitatively explored including students of all genders, including those outside the binary.

## The Current Research

Research has started to explore the relationship between camouflaging, mental health, and other factors in adult and non-autistic populations. For example, Cassidy et al. (2020) found that autistic traits predicted camouflaging, and that camouflaging was correlated with experiences of thwarted belonging, and higher suicidality. In another study, Perry et al. (2022) explored the relationship between autism-related stigma and well-being in an adult population and found that the two were associated. They also found that camouflaging was associated with stigma and well-being, however, did not mediate the relationship. They concluded that future research should explore more specific aspects of mental health.

As explained above, there is a well-evidenced link between reduced school belonging and anxiety (Arslan et al., 2020; Benner et al., 2017). Additionally, there is provisional evidence that autistic students who feel less school belonging may camouflage more (Cook et al., 2018; Myles et al., 2019), and that camouflaging is associated with increased anxiety (Cassidy et al., 2020; Beck et al., 2020; Cook et al., 2021). Together, this indicates that a mediation relationship may exist. We therefore hypothesised that camouflaging mediates the relationship between school belonging and anxiety. To our knowledge, the current research is the first explore this relationship.

It is important to understand how the school environment can change to support autistic students to be their authentic selves and experience a sense of belonging. However, most qualitative research in this area has focussed on female students only. Therefore, the current research also sought to gather student voice about what environmental factors impact their sense of belonging, and camouflaging behaviours.

## Methods

### Design

A mixed-methods approach was chosen because it can provide greater insight and knowledge about an area of interest than either methodology alone (Landrum & Garza, 2015). This is especially relevant because the chosen design allowed us to explore the relationship between belonging, camouflaging and anxiety, while gaining participant voice about the *how* and *why* (Parra et al., 2021). Further, it is important that research methods reflect the epistemological stance taken (Braun & Clarke, 2006). The mixed-methods design fits a critical realist perspective (McEvoy and Richards 2006). Critical realists believe that there is a real world that can be observed, but human experience of this is fallible due to our perceptions and biases (Guba & Lincoln, 1994).

### Participants

Participants were recruited from October 2022 to March 2023 via online and offline snowballing methods. These included, sharing on personal and university social media accounts (Facebook and Twitter), emails to local autism support groups (e.g., support, advocacy and social clubs), emails to school staff and word-of-mouth through personal contacts. Young people who completed the survey were reimbursed for their time with a shopping voucher.

A priori power calculations predicted that, on the assumption that the effect size for path a (belonging and camouflaging) will be medium (0.39) and path b (camouflaging and anxiety) will be medium (0.39), a sample size of 78 would be necessary to detect a mediating effect (Fritz & MacKinnon, 2007). Due to the limited research base, we were not sure of the strength of the path between belonging and camouflaging (path a), and therefore explored a more conservative sample size estimation; on the assumption that the effect size for path a was small (0.26) it was predicted that a sample of 126 would be necessary to predict a mediating effect (Fritz & MacKinnon, 2007). We therefore aimed to recruit 126 participants, with a minimum of 78.

The parent/carer survey was completed by 110 people, who gave consent for their children to take part. Of these, 72 autistic young people completed the survey. Demographic data is only provided of participants who completed both parts.

Both those with a diagnosis of autism, and those who self-identify as autistic were recruited. The majority of participants (83%) had a diagnosis of autism. For participants with a diagnosis of autism, the age of diagnosis ranged from 2 to 15 (*M* = 10.03, *SD* = 2.85). There was no significant difference between participants who were autistic (*M* = 7.92, *SD* = 1.61), and self-identifying (*M* = 7.86, *SD* = 1.20) on levels of autistic traits, *t*(70) = 0.12, *p* =.91, with a very small effect size (*d* = 0.04; Sawilowsky, 2009). The average score for both groups was above the recommended cut-off (six) for ‘specialist assessment’, suggesting they are autistic (Allison et al., 2012). There were no significant differences between the groups on camouflaging (*t*(70) = -0.96, *p* =.34, *d* = 0.3), sense of school belonging (*t*(70) = 0.58, *p* =.56, *d* = 0.18) and anxiety (*t*(69) = -0.55, *p* =.59, *d* = 0.17), further justifying analysing them together.

All young people who took part attended mainstream secondary schools in the UK or Ireland and were aged between 11 and 16 (*M* = 13.37, *SD* = 1.42). All participants had a reading age of at least 8, to ensure that they could complete the survey independently. Over half (54%) of the participants identified as male. Further demographic information is shown in Table 1, demonstrating that the majority (81.9%) of the sample identified as White, which is not representative of the UK population (61.3%; Department for Education, 2024).

**Table 1:** Demographic information for young people who completed the survey

Demographic information	Frequency	Percentage (%)
**Diagnosis of autism?**		
Yes	60	83.33
Self-identify	12	16.67
**If yes, who diagnosed?**		
Paediatrician	14	23.33
Psychologist	16	26.67
GP	2	3.33
Speech and language therapist	2	3.33
More than one professional	22	36.67
Other	2	3.33
Don’t know	2	3.33
**Age**		
11	6	8.33
12	17	23.61
13	17	23.61
14	12	16.67
15	16	22.22
16	4	5.55
**Gender**		
Male	39	54.17
Female	25	34.72
Non-binary	4	5.55
Other	1	1.39
Prefer not to say	3	4.17
**Biological sex at birth**		
Male	40	55.56
Female	30	41.67
Prefer not to say	2	2.78
**Ethnic group**		
White English/Welsh/Scottish/Northern Irish/British	59	81.9
White Irish	5	6.9
White and Asian	2	2.8
White and Black Caribbean	2	2.8
Other	3	4.2
Prefer not to say	1	

Note. Response percentages are rounded and therefore may not add up to 100

### Procedures

A panel of six autistic young people were consulted to ensure the research was relevant and accessible. This discussion informed updates to the methodology. These included: their preferred language; reimbursement for their time; providing definitions; layout of questions; phrasing of open-ended questions, and the information provided in the information sheet.

Parent/carers completed an online survey and provided informed consent for their child’s participation. Next, they were sent (via email) an individual link to the survey to be completed by their child. Surveys used the Qualtrics online survey platform, and parent and child surveys were linked through a randomised 4-digit code.

### Materials

#### Parental Survey - Demographic Questions

Parent/carers were asked to share an approximate percentage of their child’s school attendance, and whether they had an autism diagnosis or self-identified. If they were diagnosed, they were asked at what age, and by whom.

#### Parental Survey - Autism Quotient

To gather information and quantify the autistic traits of the young people, parents answered the Autism Quotient-10 – adolescent version (AQ-10-A; Allison et al., 2012). This questionnaire is a shortened version of the 50-item Autism Quotient (AQ) and has 10 questions that are rated on a four-point scale (definitely agree, slightly agree, slightly disagree, definitely disagree). As in previous research, answers were dichotomised into ‘agree’, and ‘disagree’ (Cassidy et al., 2020; Booth et al., 2013), and scored one if they suggested autistic traits, and zero if not. Scores could therefore range from zero to 10, with higher scores indicating higher autistic traits. A score of six or above indicates level of autistic traits that require an autism diagnostic assessment. Allison et al. (2012) found the AQ-10-A to have high accuracy; positive predictive value of autism (PPV) = 0.86, specificity = 0.95, and sensitivity = 0.93. The checklist has high internal consistency (α = 0.89).

#### Young Person Survey - Demographic Questions

Participants reported their age, gender, sex assigned at birth, and ethnicity. These questions were not compulsory, and there was the option to report ‘prefer not to say’.

#### Young Person Survey - Questionnaires

Research has found that middle response options can indicate confusion, ambivalence, or a non-response, and are inconsistently used (Hernández et al., 2004; Whiting et al., 2018). To keep answers consistent, and reduce confusion for participants, all questionnaires used the same 4-item Likert-scale (never, sometimes, often, always).

##### Adapted CAT-Q

An adapted version of Camouflaging Autistic Traits Questionnaire (CAT-Q; Hull et al., 2019) was used to measure levels of camouflaging. The original scale is a 25-item measure of self-reported camouflaging and Hull et al. (2019) found high internal consistency (α = 0.94) and test-retest validity is (*r* =.77). Halsall et al. (2021) adapted the scale for secondary aged pupils, removing repeated questions. Further, they simplified wording, included cartoons to support understanding, and used a 4-point scale. Previous research suggests camouflaging behaviours are different in home and school contexts (Cook et al., 2018; Halsall et al., 2021). I therefore further adapted Halsall et al’s. (2021) questions so they were limited to the school context e.g., “I try to be like someone else *at school*”. The finalised adapted questionnaire had 20 questions, and scores ranged from one (never) to four (always). Scores could therefore range from 20 to 80, with higher scores indicating more camouflaging behaviours.

##### Simple School Belonging Scale

The Simple School Belonging Scale (Whiting et al., 2018) is a 10-item Likert-scale questionnaire that is used to measure participants sense of school belonging, this item was chosen as it is specific to school belonging. Whiting et al. (2018), found preliminary evidence of construct validity (*r* =.64) and good internal consistency (α = 0.91). Scores could range from 10 to 40, with a higher score indicating higher sense of school belonging.

##### The Anxiety Scale for Children – Autism Spectrum Disorder (ASC-ASD)

The ASC-ASD (Rodgers et al., 2016) is a 24-item self-report questionnaire used with autistic children and young people aged 8–16. The scale explores four sub-scales of anxiety (separation anxiety; uncertainty; performance anxiety, and anxious arousal). Scoring guidelines suggest scoring from zero (never) to three (always), meaning total scores can range from zero to 72, with higher scores indicating higher levels of anxiety. A total score of 20 or above indicates significant anxiety levels. Rodgers et al. (2016) found the scale to have excellent internal consistency (α = 0.94) and one month test-retest reliability (*r* =.82). There is evidence that anxiety levels are context dependent, with the school environment being particularly anxiety inducing for autistic students (Adams et al., 2020; Hebron & Humphrey, 2014). We were therefore particularly interested in the school context, and adapted the scale accordingly, e.g., “*In school*, I worry what other people think of me”.

#### Young Person Survey - Qualitative Questions

Open-ended questions, that were based on previous research (Porter & Ingram, 2021) and developed in collaboration with young people, were asked following the CAT-Q and Simple School Belonging Scale. Participants were provided an open text box where they could write as much as they liked. See below for questions:

1. Do you feel like you can be your true self at school?

- If yes, what helps with this?
- If no, why not?

2) Feeling that you belong means feeling safe, accepted, and comfortable. Do you feel like you belong at school?

- If yes, what helps with this?
- If no, why not?

### Data Analysis

#### Quantitative Analysis

I exported all anonymised data using SPSS (Version 28). I used correlations to explore associations between included variables. The PROCESS (Version 4.2) add-on was used to complete mediation analysis, with the bootstrapping method for 5000 samples. The data met all necessary assumptions for the model, including no significant outliers, no co-linearity, and normal distribution.

#### Qualitative Analysis

Content analysis was chosen as an approach to systematically describe data, make inferences about experiences, provide new insights and guide action (Schreier, 2012; Krippendorff, 2019). I used both inductive and deductive approaches guided by existing frameworks of school belonging (Craggs & Kelly, 2018), and previous research in the area (Porter & Ingram, 2021; Hull et al., 2017; Cage & Troxell-Whitman, 2019). Each question was analysed separately. This flexible and reflective process followed three iterative stages based on Elo and Kyngäs (2008). Each stage was discussed with co-researchers, until we reached consensus.

##### Preparation

I read data several times to become immersed. I then identified meaning units, which are the smallest unit that contains data that answers the research question.

##### Organisation

I organised meaning units into codes (labels that accurately describe the meaning unit) and then grouped codes into categories. Due to the data collection method, data was not rich in latent meaning. I therefore chose categories, rather than themes, to be the highest level of abstraction, and adopted a manifest analysis approach that was guided by, and stayed close to participant data (Erlingsson & Brysiewicz, 2017).

##### Reporting

To check consistency, a second independent researcher independently sorted the meaning units into existing categories, using the coding frame (See Appendix E; Schreier, 2012; Bengtsson, 2016). Agreement levels were moderate for the belonging questions (κ = 0.79, *SE* = 0.07, *p* < .001), and strong for camouflaging questions (κ = 0.85, *SE* = 0.06, *p* < .001; McHugh, 2012).

## Results

### Quantitative Analysis

Due to the question format of the online survey, and the ethical decision to not make any questions compulsory, a small number of participants did not answer all questions. One participant ended the survey before the ASC-ASD. A further five participants missed one or two questions, for these participants, in accordance with scoring guidelines, I prorated the remaining items in the appropriate scale (Rodgers et al., 2016). Two participants had missing data from the belonging scale and AQ-10, and one from the CAT-Q. Single mean imputation using SPSS was used to replace missing values (Mirzaei et al., 2022). This did not change the overall pattern of results.

Average scores for overall measures, and sub-scales of each questionnaire are shown in Table 2. Correlations between variables are shown in Table 3. Further analysis of significant correlations showed that females (biological sex) reported higher levels of anxiety (*M* = 41.75, *SD* = 14.39) than males (biological sex; *M* = 33.30, *SD* = 12.63), *t*(67) = -2.59, *p* =.01, *d* = 0.63. Additionally, females had a lower mean for school attendance (M = 76.60, SD = 29.18) than males (M = 88.90, SD = 18.87) with equal variances not assumed, this approached significance *t*(46.64) = 2.01, *p* =.05, *d* = 0.52).

**Table 2:** Means, standard deviations and range of scores for variables in the study

	Mean (SD)	Range	Skewness*	Kurtosis*
Autism traits	7.91 (1.54)	4–10	-0.61	-0.03
Belonging	20.95 (5.63)	10–35	0.18	-0.41
Camouflaging	46.01 (9.06)	25–66	0.19	-0.45
Anxiety (Overall)	37.52 (14.31)	7–66	-0.14	-0.56
*Performance anxiety*	9.30 (3.48)	1–15	-0.10	-0.84
*Anxious arousal*	7.51 (4.35)	0–18	0.21	-0.36
*Separation anxiety*	6.62 (4.21)	0–15	0.03	-1.07
*Uncertainty*	14.10 (5.48)	3–24	-0.10	-0.76

*Scores between − 2 and + 2 are generally acceptable in normal distribution (Hair et al., 2010)

**Table 3:** Correlations between variables included in the study

	Attendance (%)	Diagnosis?	Age	Age at diagnosis	Sex	AQ-10-A	CAT-Q	ASC-ASD
Diagnosis?	0.17							
Age	-0.17	-0.17						
Age at diagnosis	-0.07	N/A	0.27*					
Sex	-0.25*	0.07	0.05	0.05				
AQ-10-A	0.02	-0.01	0.11	0.24	-0.02			
CAT-Q	-0.10	0.11	0.18	0.11	0.16	0.02		
ASC-ASD	-0.36**	0.07	0.08	0.12	0.30*	0.09	0.55**	
Belonging	0.15	-0.07	-0.09	-0.18	-0.21	-0.22	-0.45**	-0.50**

Note. *p < .05, **p < .01 level, two-tailed

#### Mediation Analysis

Previous research suggests that age, sex, and autism traits are correlated with camouflaging (Cook et al., 2021; Wood-Downie et al., 2021; Ross et al., 2023). Due to this, and indicative correlations between school attendance, anxiety, and biological sex (See Table 3), we entered the variables (age, sex, autistic traits, and school attendance) as co-variates.

**Fig. 1:**
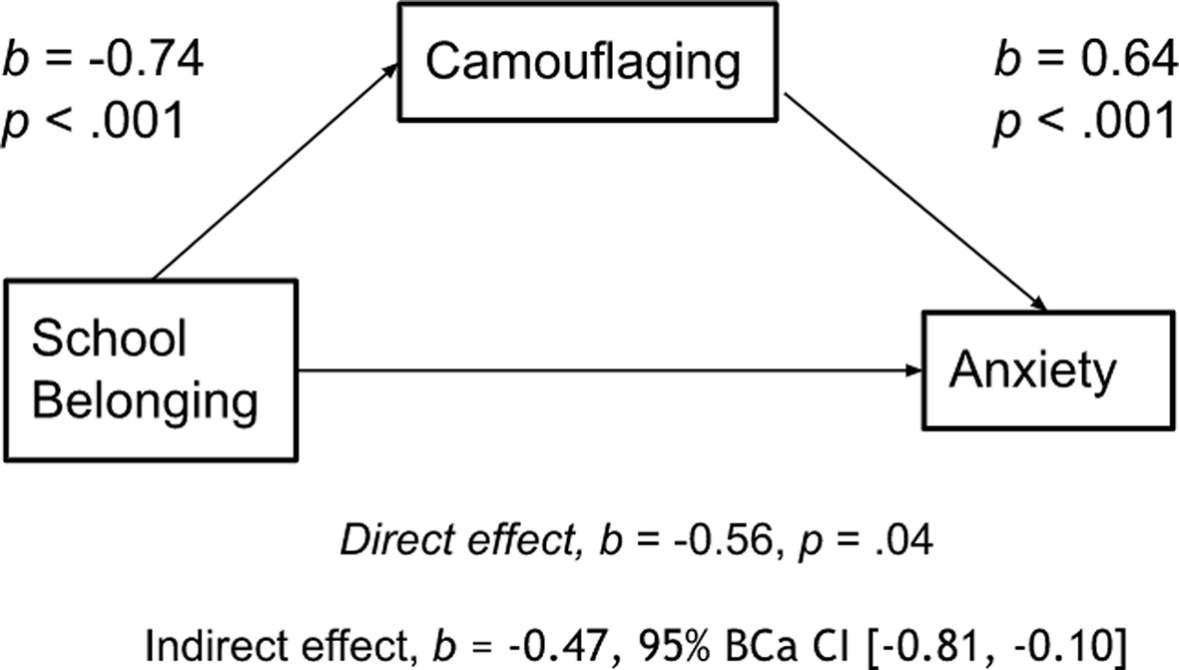
Model of belonging as a predictor of anxiety, mediated by camouflaging. *Note.* The confidence interval for the indirect effect is a BCa bootstrapped CI based on 5000 samples

The path between belonging and camouflaging was significant [*b =* -0.74, *t =* -3.94, *p* < .001], as was the path between camouflaging and anxiety [*b* = 0.64, *t =* 3.98, *p* < .001]. The direct effect between belonging and anxiety was significant [*b =* -0.56, *t* = -2.10, *p* =.04], and there was a significant indirect effect of belonging on anxiety through camouflaging, *b* = -0.47, 95% BCa CI [-0.81, -0.11]. The mediation is shown in Fig. 1.

### Qualitative Analysis

#### Belonging

Twenty-eight participants (38.9%) reported that they did feel safe, accepted and comfortable at school, and 44 (61.1%) that they did not. From the responses to open-ended questions five categories were identified (friendship and peer interactions; what I am and what I do; acceptance and understanding; school environment and structures, and the role of school staff). Additionally, a small proportion of participants were unsure, or did not answer the question. Table S1 (in the supplementary materials) shows the frequency of codes in each category. The current research focussed on the overall experience of autistic students in schools, and therefore we did not conduct separate analyses based on individual characteristics (e.g., age and gender). It is, however, noteworthy that participants of all genders, including those outside of the binary, were similarly represented across categories throughout the questions.

##### What Helps You to Feel you Belong?

Friendships and interactions with peers, were commonly cited reasons for feeling that they did belong at school. Indeed, nine participants referred to their friends or friendship group: “I have friends that would help me”, “I have cool friends”, “my friends are nice and help me fit in”. Others referred more generally to positive interactions with other students: “I like chatting to people”.

Students also commonly mentioned the ‘role of school staff’. Nine students referred to teachers who are “responsible”, “caring”, “kind” and “help me to feel safe”. A teaching assistant was mentioned by one student, and all staff by another.

The ‘school environment and structures’ were the next most cited category. Students spoke about having an inclusion hub that they could access (3). More generally, one student felt that it was a “safe environment” and others discussed school systems and routines (2).

References to identity and individual actions (who I am and what I do) were mentioned by five students, with four referring to their aptitude for school learning: “I am mostly very good at school subjects”. Finally, two students shared that others’ understanding helped them to feel a sense of belonging to school.

##### Why Do You Feel You Don’t Belong?

In contrast to the previous question, ‘who I am and what I do’ was the most mentioned category for students who did not feel a sense of school belonging. Participants referred to feeling “different” (4) and finding it harder than other people (2). One participant shared that they do not “fit in”, another that they did, but only when “I am not being my true self”, leading them to wonder: “do my friends only like the school me or the true me?”. Some referred to feeling they belonged elsewhere (2), and one that they “wouldn’t want to belong anyway”.

Next was ‘friendship and peer interactions’; participants referenced “immature” and “unfriendly” students and feeling “hated”. Additionally, social isolation was mentioned by two students: “They don’t make an effort to be my friend”, “I often feel left out and ignored”. Bullying was also mentioned by three students: “loads of kids bully me”, “I get a lot of death threats”.

Thirdly, nine participants cited ‘acceptance and understanding’ from others as negatively impacting their sense of belonging. Five mentioned a lack of understanding from others, and three that they were not accepted: “I try so hard to be accepted and people don’t want to know me”, and the final that “I feel like people are silently judging me”.

Less commonly mentioned was the ‘school environment’ (5). Three participants referred to not feeling physically safe, two discussed school processes and activities: “I am worried about all the homework and PE”, and one discussed the “loud noises”. Only three participants mentioned the ‘role of school staff’ in feeling that they did not belong. Of these, teachers were perceived as “mean”, and one student wrote “staff don’t care about my needs as long as I get good grades, even if I try to kill myself they don’t care”.

#### Camouflaging

Twenty-four participants (33.3%) said that they did feel that they can be their real self at school, and 48 (66.7%) said that they could not. Four categories were identified (environment, support and adaptations; who I am and what I do; acceptance and understanding; my social relationships). Table S2 (in the supplementary materials) shows the frequency of codes in each category.

What Helps you to be Your True Self? Eight comments referred to how social relationships supported students to feel that they could be their true self in school. Most commonly, these were friendships (6): “My friends help me to be real by making me feel loved”, “I have a good friendship group who are similar”. Additionally, two students appreciated that all students were friendly.

The ‘environment, support and adaptations’ supported some young people to be their real self. They referenced staff support (3), and a range of adjustments that helped them including: wearing headphones (3), access to a quiet room, an inclusion hub, and using their iPhone.

Thirdly, there were six comments that referred to ‘who I am and what I do’. Some had a positive view of who they were: “who I am is cool”, “I like being myself”. For others it was their skills such as “good communication” that helped them. One participant made an effort to be themselves through “practising”. Finally, only two comments referred to acceptance and understanding: “me knowing that most people won’t judge me”.

##### What Makes it Hard to be Your True Self in School?

A large proportion of comments made (67%) referred to ‘acceptance and understanding’ from others as a reason that they could not be their true self in school. Often, students were fearful of other people’s reactions to them being their real self, feeling that they will be disliked (4), perceived as weird (5), and judged (6). Eleven participants mentioned being bullied, or laughed at (3), if they were to be their real self. For some this was due to observing their peers’ behaviours: “Most people at my school would most likely bully me, just the way life is”, and for others, a fear that history would repeat itself “I was severely bullied at my last school and know it will happen again if I don’t mask”.

The next most cited category was ‘who I am and what I do’. Some students felt that who they are, and their difference meant that they could not be their true self (5): “I feel like myself is not an okay person to be”. Two found it “too hard” and one that it is “easier to hide my difficulties”. Difficulties with identity were also cited: “I don’t really know who I am”.

Just three participants referenced ‘environment, support and adaptations’. Two shared that the environment did not make them feel comfortable, and another that “the teachers have to concentrate on disciplining naughty children, rather than helping me be confident in who I am”. There were two mentions about social relationships related to feeling that other people being unfriendly and they feel they “can’t trust”.

## Discussion

The quantitative aspect of this study aimed to test, for the first time, the hypothesis that the relationship between sense of school belonging, and anxiety is mediated by camouflaging, in secondary aged autistic students. As predicted based on previous research, it was found that a significant mediation relationship did exist, even after accounting for the effects of age, biological sex, school attendance and autistic traits. This supports qualitative research suggesting low feelings of school belonging mean that autistic students hide their autistic traits at school, which increases feelings of anxiety (Porter & Ingram, 2021; Myles et al., 2019). Additionally, research in adult populations has found that camouflaging can act as a response to autism related stigma (Perry et al., 2022). The current research supports this idea, as students who felt excluded (demonstrated by low scores on belonging) were more likely to camouflage, although this needs further exploration. The findings reinforce the importance of school belonging as a protective factor against internalising symptoms (Benner et al., 2017; Arslan et al., 2020) and camouflaging as detrimental to the mental health of autistic students (Cook et al., 2021; Halsall et al., 2021).

Within the analysis there were further findings of note. For example, in contrast to previous research, we did not find a significant correlation between autistic traits or biological sex in camouflaging behaviours (Cook et al., 2021; Cassidy et al., 2020; Wood-Downie et al., 2021). In the current research, autistic trait measures were completed by parents on behalf of their children. In contrast, the majority of previous research investigating the association between autistic traits and camouflaging has been self-reported by autistic adults (Cook et al., 2021), which may account for the discrepant findings. For example, it is possible that the association between camouflaging and autistic traits increases with age, which would be a useful avenue for future research. These results also indicate that adolescent autistic males may camouflage more than initially postulated (Ross et al., 2023). Perhaps of more importance, these findings suggest that it is environmental and relational factors rather than within-person factors that influence camouflaging within the school context.

Despite biological sex not being significantly related to camouflaging, females reported higher levels of anxiety than males. Whilst research has confirmed that females in neurotypical populations have higher anxiety levels (Lewinsohn et al., 2022; Copeland et al., 2014), this has not been consistently found in the autistic population (Ambrose et al., 2020). In the current research, it is possible that the intersection between being autistic and female in the school environment is particularly anxiety inducing, impacting school attendance. Further research is needed to explore this relationship.

In the qualitative aspect of the research, 61% of participants felt that they did not belong at school, and 66% felt they could not be their real self. Given the importance of both aspects on mental health, research aimed to further understand what makes the difference, including open-ended questions about camouflaging and belonging. As the concepts of belonging, and camouflaging are related, there was much overlap between answers given to each question, though there were also distinct aspects to the responses (see results for full breakdown and the differences observed). Overall, categories related to: individual qualities and actions; school environment and structures; relationships with others, and acceptance and understanding. Previous research has found that male and female participants camouflage for different reasons (Cage & Troxell-Whitman, 2019). While the current research did not comprehensively explore this, it appeared that students of all genders were similarly represented in categories.

### Individual Factors

Personal and individual factors were mentioned by participants in all qualitative questions and have been found as a key factor associated with belonging (Allen & Kern, 2017). It was often the young persons’ perception of themselves that meant that they felt that they could or could not be their true self, and impacted their sense of school belonging. Feeling they could do well academically was positive, however, when students perceived that staff only cared about their grades it reduced their feelings of belonging. This supports the idea that schools should provide an environment that “goes well beyond a singular focus on academic attainment” (Porter & Ingram, 2021, p. 73). Often students referred to feeling “too different” or that they did not “fit in”, perhaps indicating that they had internalised stigma and the school’s neurotypical expectations (Mesa & Hamilton, 2022; Milton, 2012). Further, students shared difficulties understanding their own identity, possibly impacted by camouflaging (Hull et al., 2017). These difficulties caused anxiety and questions around the authenticity of their relationships.

### Relationships

A key element to supporting sense of belonging was relationships. As has been found in neurotypical populations, school staff were mentioned to be important in supporting a sense of school belonging (Allen & Kern, 2017). Students referred to the qualities of their teachers (kind and responsible), and it was help with staying safe, rather than academic support that was appreciated. Similarly, for participants of all genders, friendship was seen as a key aspect in what helps them to feel safe at school, extending work in female autistic populations (Halsall et al., 2021; Porter & Ingram, 2021; Cook et al., 2018). It was not clear whether the students’ friends were also neurodivergent, however, there is evidence that feeling connected to the autistic community is related to higher sense of belonging (Cage et al., 2022).

Social relationships were also key to supporting autistic students to feel that they can be their real self. Previous research has found that autistic students benefit from friendships that are based on companionship, and similarity (Cook et al., 2018). This was echoed in the current research, where students shared that they liked having friends who were similar. According to the ‘double empathy problem’ breakdowns in communication between autistic and non-autistic people occur due to differences in social expectations and experiences of the world (Milton, 2012). It is likely that in the current research, participants favoured similar peers, as they had a shared understanding, and therefore less need to conform, resulting in authentic and positive communication (Heasman & Gillespie, 2019; Milton, 2012).

While relationships were protective, students also shared that a lack of safe relationships contributed to reduced feelings of belonging and increased camouflaging, suggesting that social isolation can act as a risk factor (Porter & Ingram, 2021).

### The School Environment

As has been found in previous conceptions of school belonging (Craggs & Kelly, 2018) a safe school environment was important to feeling a sense of belonging. For some, the sensory environment was overwhelming, and others felt physically threatened by peers (Horgan et al., 2022). Safety could be found through small changes such as allowing students to wear headphones or allowing them access to technology. Previous research has suggested that autistic students need spaces where they can be their authentic self and take off the mask (Cage & Troxell-Whitman, 2019). This was reflected in the current research, where students benefited from having access to an inclusion unit or ‘hub’ as helping them to feel they can be their true self.

### Acceptance and Understanding

Acceptance and understanding have been found to be key in developing a sense of belonging (Craggs & Kelly, 2018; Allen & Kern, 2017). A few participants in the current research shared that they felt understood by others, however, this was rare. Although there is an increasing public awareness of autism, it is well-evidenced that autistic people still feel misunderstood, indicating a greater push for education about autism is needed (APPGA, 2019; Mesa & Hamilton, 2022).

Of particular note, a lack of acceptance and understanding was cited as a reason by over 2/3 of students who felt they could not be their true self at school. Autistic students are particularly vulnerable to bullying (Cook et al., 2018; Horgan et al., 2022), and participants feared the responses of their peers when considering whether they needed to camouflage. This supports research suggesting that camouflaging is a survival strategy, and a response to stigma (Halsall et al., 2021; Hull et al., 2017; Tierney et al., 2016; Cook et al., 2018). While autistic students know that camouflaging is exhausting, they are required to weigh up the impact of camouflaging on their mental health, with the likelihood of bullying (Cage & Troxell-Whitman, 2019). This perhaps explains why one participant reported that it is easier to hide their true self.

### Implications

The current research suggests that an effective way to reduce anxiety in autistic students is to create school environments where they can experience belongingness, and therefore do not need to camouflage (Cage et al., 2022; Lawson, 2020).

Positive relationships with both staff and peers were important in supporting students to feel a sense of school belonging. Teachers often report feeling ill-equipped to support autistic students (Ravet, 2018). The current research suggests that simple steps such as showing kindness, empathy, and focusing on the person rather than the grades can make a big difference. Many students shared that they enjoyed having friends with similar interests, and joining clubs based on this could help to foster a greater sense of school belonging (Mesa & Hamilton, 2022).

In the present study, many participants referred to having access to a ‘hub’ throughout the day. Hubs appear to offer a break from the sensory and social demands of the mainstream environment allowing students a space where they can be themselves. The provision of such spaces by schools to use throughout the days is important, although arguably of more importance, is a will to make the whole environment feel safer.

The current research clearly demonstrates the importance of acceptance and understanding from other people in supporting autistic people to feel they can belong and be their true selves. It is important that schools foster an environment of understanding neurodivergence as a *difference* rather than a *disability*. It has been suggested that assemblies, Personal, Social, Health and Economic (PSHE) lessons, and events could be utilised to support peer acceptance and understanding, and reduce bullying (Mesa & Hamilton, 2022). Further, there has been preliminary evidence of the efficacy of an anti-stigma programme in secondary schools (Ranson & Byrne, 2014). Increasing awareness is likely to reduce the ‘double empathy problem’, and therefore the internalised stigma that autistic students experience (Cage et al., 2022; Milton, 2012). Researchers at York St John’s University are currently working on a project (Autism Inclusive Mainstream Secondary Schooling; AIMSS) developing an intervention to support more inclusive and understanding secondary school environments (Mesa & Hamilton, 2022), which our findings suggest could be an effective intervention in reducing camouflaging and, in turn, anxiety.

### Strengths, Limitations and Future Research

The current research is the first to explore the relationship between sense of school belonging, camouflaging, and anxiety in autistic students at mainstream schools quantitatively. We felt it was important to include participants who self-identified as autistic, given some young people may have not received a diagnosis due to their camouflaging, and would otherwise be excluded (National Association for Special Educational Needs; NASEN, 2016). Additionally, the use of an online survey meant that participants could share ideas that they may not usually due to their camouflaging (Porter & Ingram, 2021). Participants were included regardless of gender, and a small number identified as non-binary. Previous research has found that gender-divergent youth are at higher risk of mental health difficulties (Gallagher & Axelrad, 2023), with some hiding their authentic selves in order to fit into societal expectations and belong (Toft et al., 2019; Bornstein, 2022). Further research could explore the intersection of gender identity and autism, with regards to belonging, camouflaging, and mental health.

Despite the strengths, there were several limitations to the research. Despite being almost powered to detect small effect sizes based on a priori power calculations (and observed effects sizes being medium-to-large), the sample was relatively small. Therefore, replications with larger samples are needed to further substantiate conclusions. Further, the results are not generalisable to all of those on the autism spectrum and, given the recruitment strategy, likely skewed to students who had higher levels of camouflaging, or a more challenging time at school. There are issues with ethnic diversity in autism research (Steinbrenner et al., 2022), and the current research predominantly included individuals who identify as White. Participants who had difficulties with literacy were ineligible to participate in the online survey, which highlights a limitation in autism research as a whole (Fayette & Bond, 2018). Consequently, the findings of the study may not be applicable to individuals in this particular group. Further research is needed to develop more inclusive methods to test the mediation model in more diverse samples.

The current study involved self-report questionnaires via an online survey. Due to the correlational nature, directionality cannot be inferred, although qualitative accounts do provide some evidence for the direction discussed. Parents completed the AQ-10, and it is possible that their responses were skewed by parental autistic traits or expectations (Ross et al., 2023). Additionally, the CAT-Q measures the intent to camouflaging, rather than level of camouflaging employed (Cook et al., 2021). It is possible that some students may not have been conscious of their camouflaging, and discrepancy approaches could be used to further explore the relationship between actual levels of camouflaging, school belonging and anxiety.

Questionnaires are “imperfect devices” for gathering qualitative data (Porter & Ingram, 2021, p. 73). Despite co-creating questions with autistic students, it is possible that students took different interpretations of the open-ended questions, and without follow up questions it is difficult to ensure understanding. Additionally, due to the binary nature of questions, we may have missed some valuable data. Further, the strong overlap between answers given to camouflaging and belonging open-ended questions was noted, and perhaps due to unclear wording. The correlation analysis demonstrated that, although related, they are two distinct constructs, and qualitative answers show some uniqueness. It was therefore decided they would be analysed separately. The current research provides insight into the environmental influences of belonging and camouflaging, but in order to support interpretations, more research is needed with an alternative methodology (e.g., semi-structured interviews).

Finally, the scope of the current research was limited due to the measures that were included. The findings only explain some of the picture, and it is important that future researchers include variables such as disclosure, membership to autistic community, and perceived stigma (Perry et al., 2022; Cage et al., 2022; Cage & Troxell-Whitman, 2020). Despite the limitations, the results provide novel and important findings emphasising the importance of sense of school belonging for autistic students in schools.

## Conclusion

In conclusion, this mixed-method study provides evidence that, for autistic students in mainstream secondary schools, there is a relationship between sense of school belonging and anxiety, which is mediated by camouflaging. Autistic students shared that social relationships, levels of acceptance and understanding from others, safety of the environment, and their view of self were factors that either helped or hindered their sense of belonging and ability to be their true self. These findings suggest that is important to develop school cultures that foster belonging, celebrate neurodiversity, support students to have meaningful friendships, and provide safe spaces so that students can *take off the mask.*

## Electronic supplementary material

Below is the link to the electronic supplementary material.

Supplementary Material 1
