# Supplementary material for: “Do My Friends Only Like the School Me or the True Me?”: School Belonging, Camouflaging, and Anxiety in Autistic Students
Source: J Autism Dev Disord. 2025 Jan 8;56(6):2231–45. doi: 10.1007/s10803-024-06668-w (PMC13222283; doi:10.1007/s10803-024-06668-w)
Supplement: Supplementary file 1 — Supplementary Material 1 [file 10803_2024_6668_MOESM1_ESM.docx]

**Supplementary Materials**

**Table S1**

*Frequency of Codes in Categories Answering Open Ended Questions About Belonging*

| Questions  Codes | What helps you to feel belong?  *n* = 42 | | Why do you feel like you do not belong?  *n* = 49 | | Total  *n* = 91 | |
| --- | --- | --- | --- | --- | --- | --- |
|  | *n* | *%* | *n* | *%* | *n* | *%* |
| Friendship and peer interactions | 12 | 28.57 | 14 | 28.57 | 26 | 28.57 |
| Who I am and what I do | 5 | 11.90 | 15 | 30.61 | 20 | 21.98 |
| Acceptance and understanding | 2 | 4.76 | 9 | 18.37 | 11 | 12.09 |
| School environment and structures | 7 | 16.67 | 5 | 10.20 | 12 | 13.19 |
| Role of school staff | 12 | 28.57 | 3 | 6.12 | 15 | 16.48 |
| Unsure | 4 | 9.52 | 3 | 6.12 | 7 | 7.69 |

*Note. n* refers to codes, rather than participants, as some participants cited more than one reason for their answer.

**Table S2**

*Frequency of Codes in Categories Answering Open Ended Questions About Camouflaging*

| Questions  Codes | What helps you to be your real self?  *n* = 24 | | Why do you feel you cannot be your real self?  *n* = 54 | | Total  *n* = 78 | |
| --- | --- | --- | --- | --- | --- | --- |
|  | *n* | *%* | *n* | *%* | *n* | *%* |
| Who I am and what I do | 6 | 25 | 11 | 20.37 | 17 | 21.79 |
| Environment, support and adaptations | 6 | 25 | 3 | 5.56 | 9 | 11.54 |
| My social relationships | 8 | 33.33 | 2 | 3.70 | 10 | 12.82 |
| Acceptance and understanding | 2 | 8.33 | 36 | 66.67 | 38 | 48.72 |
| Unsure | 2 | 8.33 | 2 | 3.70 | 4 | 5.13 |

*Note. n* refers to codes, rather than participants, as some participants cited more than one reason for their answer.
